# Interconnected factors in EFL engagement: classroom climate, growth mindset, and achievement goals

**DOI:** 10.3389/fpsyg.2024.1353360

**Published:** 2024-10-28

**Authors:** Weiran Ma, Weian Yang, Qinggang Bu

**Affiliations:** ^1^Faculty of Education, Northeast Normal University, Changchun, Jilin, China; ^2^Education and Research Department, Primary School Attached to Northeast Normal University, Changchun, Jilin, China

**Keywords:** classroom climate, growth mindset, achievement goal orientation, student engagement, EFL education, structural equation modeling

## Abstract

**Introduction:**

This study explores the relationships among classroom climate, growth mindset, achievement goal orientation, and student engagement in the context of English as a foreign language (EFL) education in China. The study aims to understand how these factors interact to influence student engagement and motivation in EFL learning.

**Methods:**

Data were collected through a questionnaire administered to 587 Chinese undergraduate EFL students. The questionnaire assessed students’ perceptions of classroom climate, growth mindset, achievement goal orientation, and engagement. Structural equation modeling (SEM) was utilized to examine the relationships among these variables.

**Results:**

The SEM analysis revealed significant positive correlations among classroom climate, growth mindset, achievement goal orientation, and student engagement. Both classroom climate and growth mindset were found to directly and positively predict student engagement. Additionally, achievement goal orientation mediated the relationships between both classroom climate and student engagement, and between growth mindset and student engagement.

**Discussion:**

The findings underscore the interconnectedness of classroom climate, growth mindset, and achievement goal orientation in shaping student engagement in EFL education. These results suggest that fostering a positive classroom climate and promoting mastery-oriented goals can enhance student motivation and contribute to more effective language acquisition. Practical implications for EFL educators are also discussed.

## Introduction

In the dynamic realm of education, the cultivation of student engagement serves as a cornerstone for effective learning experiences (Tao et al., 2022). Engaged learners transcend passive reception of knowledge; they actively immerse themselves in their educational endeavors, displaying eagerness, inquisitiveness, and a profound dedication to learning (Fredricks et al., 2016; Lei et al., 2018). Unraveling the elements driving student engagement has become a pivotal pursuit, commanding attention from both educators and researchers (Konold et al., 2018; Trowler, 2010). Within the context of English as a Foreign Language (EFL) education, cultivating student engagement holds particular significance, as it can help learners overcome the linguistic and cultural barriers inherent in acquiring a second language (Derakhshan and Fathi, 2023; Li, 2023; Mercer, 2019; Mercer and Dörnyei, 2020; Mystkowska-Wiertelak, 2022; Wang and Wang, 2024).

Among these influential factors, the concept of a “growth mindset” has emerged prominently. Embracing a growth mindset involves believing that one’s abilities and intelligence are not fixed traits but can be nurtured and expanded through dedicated effort, continual learning, and resilience (Dweck, 2006, 2015). Individuals embodying a growth mindset embrace challenges, view effort as a stepping stone to mastery, and persist amidst adversities (Blackwell et al., 2007; Burnette et al., 2023; Yeager and Dweck, 2020). This perspective holds profound implications for education, fostering a passion for learning and resilience when facing challenges in various contexts (Bai and Wang, 2020; Dweck, 2006; Fathi et al., 2024a; Macnamara and Burgoyne, 2023).

Additionally, classroom climate, a microcosm of the broader school environment, significantly shapes student experiences, motivation, and engagement within the various educational contexts (Fraser, 1989; Joe et al., 2017; Wang et al., 2020). It encompasses the quality of teacher-student relationships, peer interactions, and the emotional and academic support that students receive (Marsh et al., 2012; Tu, 2021; Wang and Holcombe, 2010). Research consistently demonstrates the profound impact of classroom climate on both the cognitive and affective domains in EFL contexts (e.g., Liu, 2023; Liu and Geng, 2023; Mohammad Hosseini et al., 2022; Namaziandost et al., 2024; Yang and Lin, 2024), making it a critical area of focus for language educators and researchers. Moreover, students’ achievement goal orientations significantly shape their engagement levels (Miller et al., 2021). According to Achievement Goal Theory (Ames, 1992), students pursue varied educational goals, such as mastery goals (focused on learning and skill development) or performance goals (focused on demonstrating competence compared to peers). These pursuits profoundly influence students’ attitudes and behaviors within educational settings (Baranik et al., 2010; Bardach et al., 2020; Bai and Wang, 2020; Jiang and Zhang, 2021; Liu et al., 2023; Wang et al., 2023).

Despite the wealth of research on student engagement, growth mindset, classroom climate, and achievement goal orientation, a significant gap remains in understanding how these constructs interact to influence EFL learners’ engagement. Specifically, while growth mindset and classroom climate have been studied independently, there is limited research examining their combined effects on EFL student engagement. Additionally, the role of achievement goal orientation as a potential mediator in these relationships is yet to be fully explored, particularly in EFL contexts where language learning presents unique challenges and demands.

The purpose of this study is to investigate the interconnected relationships among growth mindset, classroom climate, achievement goal orientation, and student engagement in EFL learners. By examining how these psychological and environmental factors interact, the study aims to fill the gap in existing research by providing a more comprehensive understanding of the dynamics that shape student engagement in EFL contexts. Specifically, this study explores the direct and indirect effects of growth mindset and classroom climate on student engagement, with achievement goal orientation serving as a mediating variable.

The significance of this study lies in its potential to offer practical insights for educators and policymakers aiming to enhance EFL learners’ engagement and academic success. By identifying the key factors that contribute to sustained engagement, this research can inform the development of targeted interventions that foster a positive classroom climate, promote mastery-oriented goals, and cultivate a growth mindset in EFL learners. Moreover, the findings may provide a framework for future research exploring the broader implications of these constructs in diverse educational contexts, thereby contributing to the advancement of both theory and practice in the field of language education.

## Literature review

### Growth mindset and student engagement in EFL learning

Student engagement, encompassing behavioral, emotional, and cognitive dimensions, refers to students’ active participation and commitment to learning (Fredricks et al., 2004, 2016; Tao et al., 2022). Behavioral engagement involves involvement in classroom activities and adherence to classroom rules (Fredricks et al., 2004). Emotional engagement includes feelings like enjoyment or anxiety related to learning (Parsons and Taylor, 2011; Reeve, 2012; Roorda et al., 2017). Cognitive engagement pertains to deep thinking, the use of learning strategies, and self-regulation (Oga-Baldwin, 2019; Sinatra et al., 2015). These dimensions are interconnected; emotional engagement often supports behavioral and cognitive engagement, leading to improved academic performance and well-being (Fredricks et al., 2016; Wang and Eccles, 2013).

In the context of second language acquisition (SLA), student engagement is crucial for successful language learning (Mercer, 2019; Mystkowska-Wiertelak, 2022). Engagement in EFL involves not only emotional and behavioral participation but also cultural interactions and cognitive processing (Jiang et al., 2021). Factors such as a supportive classroom environment with clear expectations (Hoi, 2022), teachers who are warm and approachable (Hu and Wang, 2023), and strong teacher-student relationships (Gan, 2021) have been identified as important for enhancing student engagement in EFL contexts. Additionally, individual psychological traits like a growth mindset play a significant role in fostering engagement.

A growth mindset—the belief that intelligence and abilities can be developed through effort and learning—encourages a resilient approach to challenges and views setbacks as opportunities for improvement (Dweck, 2006; Dweck and Leggett, 1988; Burnette et al., 2023). Research shows that individuals with a growth mindset are more likely to embrace challenges, practice self-regulation, and focus on mastery-oriented goals (Claro et al., 2016; Rhew et al., 2018). These characteristics are linked to greater resilience, active engagement, and better academic performance (Mueller and Dweck, 1998; Sisk et al., 2018).

In EFL learning, a growth mindset is considered essential for fostering student engagement and resilience (Derakhshan et al., 2022; Fathi et al., 2024b; He et al., 2023; Li, 2023). Students with a growth mindset are more likely to set mastery-oriented goals, engage in self-directed learning, and exhibit higher levels of intrinsic motivation—all crucial for language acquisition (Bai and Guo, 2019; Bai et al., 2020). Moreover, a growth mindset helps mitigate negative emotions like boredom and frustration, which can hinder engagement when facing language learning challenges (Derakhshan et al., 2022). By perceiving challenges as opportunities for personal growth, these students tend to stay positive, seek additional learning opportunities, and actively participate in language tasks (Bai and Wang, 2023; Tseng et al., 2020; Zeng et al., 2016; Zhao et al., 2021). This approach fosters the resilience needed to persist through the complexities of learning a new language, leading to sustained engagement over time.

Supportive teacher-student relationships further enhance the benefits of a growth mindset by creating a learning environment where students feel valued, safe to take risks, and motivated to participate actively (Li, 2023). Given these insights, it is reasonable to expect that fostering a growth mindset will lead to higher levels of student engagement in EFL learners.

*Hypothesis 1*: Growth mindset is positively associated with student engagement in EFL learners.

### Classroom climate and EFL engagement

Classroom climate, broadly defined as the emotional and academic atmosphere within a learning environment, is a key determinant of student motivation, engagement, and overall experiences (Fraser, 1989; Joe et al., 2017; Wang et al., 2020). A positive classroom climate, where mutual respect, teacher support, and collaboration are prioritized, fosters intrinsic motivation and contributes to improved academic outcomes (Alonso-Tapia and Ruiz-Díaz, 2022; Patrick et al., 2011; Reeve and Tseng, 2011; Wang and Degol, 2016). This becomes particularly significant in EFL contexts, where learners often navigate challenges related to language acquisition, such as anxiety, self-doubt, and cultural adjustment (Liu, 2023; Liu and Geng, 2023; Qiu, 2022).

The connection between classroom climate and student engagement is well-documented. Research indicates that students who perceive their teachers as supportive and their learning environment as collaborative are more likely to demonstrate higher levels of engagement and motivation (Lombardi et al., 2019; Martin and Rimm-Kaufman, 2015; Roorda et al., 2017; Thijs and Fleischmann, 2015). Central to this dynamic are teacher-student interactions. When teachers set clear expectations and provide emotional support, students feel empowered to engage deeply with learning materials. This creates a classroom atmosphere where students feel safe to ask questions, take risks, and participate in language tasks (Li, 2023; Longobardi et al., 2019; Rimm-Kaufman et al., 2009; Shi et al., 2023). Notably, when teachers emphasize mastery goals—focused on personal growth and improvement rather than competition—students are more likely to develop intrinsic motivation, which is a powerful driver of engagement (Ames, 1992; Fredricks et al., 2004; Skinner and Pitzer, 2012).

In EFL settings, the role of a positive classroom climate becomes even more critical. The complexities of language learning can often trigger heightened anxiety or frustration among learners. However, studies have shown that a supportive classroom environment can alleviate these negative emotions, fostering psychological safety and promoting active participation (Liu and Geng, 2023; Stroet et al., 2013). When learners feel safe and respected within their learning environment, they are more willing to engage in communicative activities and participate in language tasks. This directly impacts their success in language acquisition, enhancing not only engagement but also academic achievement (Mohammad Hosseini et al., 2022).

The benefits of a positive classroom climate extend beyond mitigating anxiety and promoting participation. A supportive learning environment has been shown to enhance Foreign Language Enjoyment (FLE), a key affective factor in EFL learning. FLE refers to the enjoyment learners derive from the process of acquiring a new language, and it has been linked to increased engagement and persistence through challenges (Liu and Geng, 2023; Wang and Wang, 2024). The interaction between cognitive and affective factors—such as a positive classroom climate and emotional intelligence—not only bolsters short-term engagement but also contributes to long-term communicative competence and overall academic success (Namaziandost et al., 2024; Yang and Lin, 2024). Thus, based on the literature reviewed, this study hypothesizes:

*Hypothesis 2*: Classroom climate is positively related to student engagement in EFL learners.

### The mediating role of achievement goal orientations

Achievement goal orientation, a key concept in educational psychology, significantly impacts students’ learning behaviors and outcomes (Button et al., 1996; Pastor et al., 2007; Putwain et al., 2018). This construct reflects students’ beliefs about their abilities, which are shaped by factors such as their sense of security and the quality of relationships with teachers (Muis and Edwards, 2009; Rusk and Rothbaum, 2010). When students experience supportive teacher-student relationships and feel a sense of competence in the classroom, they are more likely to develop a positive achievement goal orientation, leading to increased motivation and academic success (Erdem and Kaya, 2024; Lamb, 2017; Ryan and Deci, 2000; Walker and Graham, 2021).

Achievement goal orientation can be divided into two main types: mastery and performance. Mastery-oriented individuals prioritize personal growth, skill development, and learning, even when faced with challenges (Midgley et al., 1998). In contrast, performance-oriented individuals are more focused on external validation, such as receiving praise or avoiding negative judgment, and may be more likely to avoid challenging tasks (Howell and Watson, 2007; Sackett et al., 2017). In the context of EFL learning, mastery-oriented students are generally more engaged with language tasks, showing resilience and persistence, which are crucial for success (Cheng, 2023; Bai and Wang, 2023; Izadpanah, 2023). On the other hand, performance-oriented students may hesitate to participate in communicative tasks due to fear of failure, limiting their engagement (Sackett et al., 2017).

There is a well-established connection between growth mindset and mastery-oriented goals. Students who believe in the potential for growth and improvement in their language abilities are more likely to adopt mastery goals, which emphasize learning and development over external validation (Claro et al., 2016; Derakhshan and Fathi, 2024). This growth mindset, when combined with mastery-oriented goals, fosters a deep engagement with learning, as well as resilience in overcoming challenges common to EFL learners (Bai and Wang, 2023; Dweck et al., 1995). Moreover, achievement goal orientations act as a mediator between growth mindset and engagement. Mastery goals encourage the use of self-regulated learning strategies and help students see setbacks as opportunities for growth. This mindset amplifies the positive effects of growth mindset on student engagement, leading to sustained involvement in academic tasks (Wolters and Hussain, 2015; Alhadabi and Karpinski, 2020). Hence, we propose the following hypothesis:

*Hypothesis 3*: Achievement goal orientations mediate the relationship between growth mindset and student engagement in EFL learners.

Similarly, a positive classroom climate can promote the adoption of mastery-oriented goals. In EFL settings, where learners often struggle with anxiety or self-doubt, a supportive environment that emphasizes personal growth and self-improvement over external validation can reduce these negative feelings and foster engagement (Jiang and Zhang, 2021; Liu and Geng, 2023). When students feel supported, they are more likely to prioritize learning and skill development, which drives higher levels of engagement and persistence (Huang, 2016; Mohammad Hosseini et al., 2022; Senko and Miles, 2008). Research indicates that a mastery-oriented classroom fosters collaboration and active participation, encouraging students to take risks, engage in communicative activities, and persist through difficulties (Feng et al., 2023; Liu et al., 2023). Thus, it is proposed:

*Hypothesis 4*: Achievement goal orientations mediate the relationship between classroom climate and student engagement in EFL learners.

In conclusion, this study highlights the role of achievement goal orientations as a key mediator linking individual factors (such as growth mindset) and contextual factors (such as classroom climate) to student engagement in EFL learning. By exploring how achievement goals mediate these relationships, we gain a clearer understanding of the mechanisms that drive motivation and engagement in language learning. This approach aligns with Wen et al.'s (2024) “Mediated Effects” principle, which explains how the impact of one variable on another can be partly attributed to an intermediary factor. Additionally, this research investigates the combined influence of individual and contextual factors on student engagement, consistent with Wen et al.’s (2024) focus on examining “Multiple Influence Factors” in complex educational settings.

### Theoretical model

**Figure 1:**
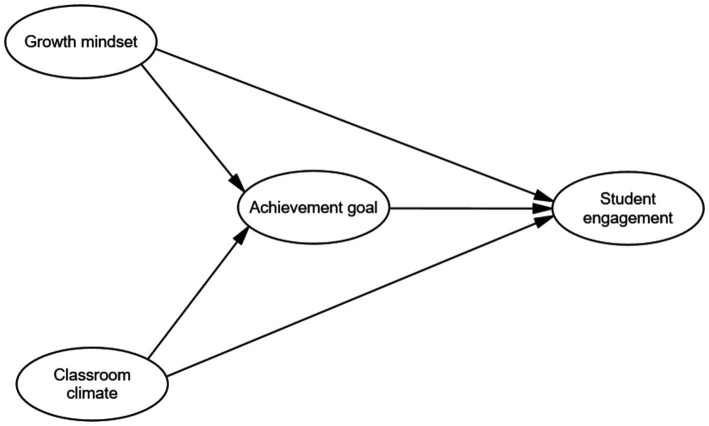
The hypothesized model.

Based on the literature and the hypotheses outlined, we propose a theoretical model illustrating the relationships among growth mindset, classroom climate, achievement goal orientations, and student engagement in EFL learners. As depicted in Figure 1, both growth mindset and classroom climate are expected to have direct positive effects on student engagement (Hypotheses 1 and 2). Additionally, achievement goal orientations are hypothesized to mediate the relationships between growth mindset and student engagement (Hypothesis 3), and between classroom climate and student engagement (Hypothesis 4). This model integrates individual psychological factors and environmental influences to provide a comprehensive understanding of what drives student engagement in EFL contexts.

By presenting this theoretical model, we aim to highlight how individual beliefs and perceptions of the learning environment interact to influence engagement. Understanding these relationships can inform educators and policymakers in developing strategies that foster a growth mindset, create positive classroom climates, and promote mastery-oriented goals, ultimately enhancing student engagement and success in language learning.

## Methods

### Participants and procedures

The participant cohort for this study comprised 587 undergraduate students majoring in English at a university in Nanjing, Jiangsu province. These students were enrolled in various English-related academic programs, such as English language and literature, translation studies, and applied linguistics, which are commonly offered as part of the English majors at Chinese universities. Their ages ranged from 18 to 24 years old (*M* = 20.5, SD = 1.8). Among the participants, 12.9% (*n* = 76) were 18 years old, 28.6% (*n* = 167) were 19 years old, 35.4% (*n* = 208) were 20 years old, 18.2% (*n* = 107) were 21 years old, 4.3% (*n* = 25) were 22 years old, and 0.6% (*n* = 3) were 23 years old, while 0.1% (*n* = 1) were 24 years old at the time of data collection. Regarding gender distribution, 47.9% (*n* = 281) were male, and 52.1% (*n* = 306) were female students.

This study was conducted as part of an exploration into non-cognitive factors’ influence on academic achievement among university students. Ethical approval was obtained from the Ethics Committee of the author’s university before the commencement of the study, adhering to the guidelines and regulations set forth by the university’s research protocols. Data collection was carried out over an 8-day period, integrated into the regular academic semester. Trained administrators oversaw the entire process to ensure procedural consistency and ethical compliance. Students voluntarily participated in the study and were assembled in designated spaces equipped with computer facilities to facilitate questionnaire completion. Comprehensive instructions were provided to encourage thoughtful and genuine responses. Participants were guaranteed anonymity and confidentiality in their responses to foster open and honest feedback. On average, students spent approximately 25 min to complete the questionnaire.

### Instruments

#### Achievement goal orientation

To assess students’ achievement goal orientation, the Achievement Goal Oriented Questionnaire originally developed by Button et al. (1996) and subsequently translated and revised by Xu et al. (2000) was adopted. This instrument consists of 12 questions in total, each addressing facets of achievement goals and mastery goals. Participants responded to the items using a 5-point scale ranging from 1 (very inconsistent) to 5 (very consistent). A sample item is “I am willing to take tasks that impart me new knowledge.” The questionnaire’s average scores were computed, with higher scores indicative of a stronger orientation toward achievement goals. The questionnaire’s reliability and validity have been well-established in the context of Chinese college students (Xu et al., 2000). In the current study, Cronbach’s alpha for this questionnaire was 0.82, indicating satisfactory internal consistency.

#### Classroom climate

The assessment of the participants’ perceptions regarding the classroom climate relied on a scale developed by Joe et al. (2017). This comprehensive scale encompasses three primary aspects, each demonstrating commendable reliability coefficients as reported by Joe et al. (2017): Teacher’s Academic Assistance consisting of 3 elements with an internal consistency coefficient of *α* = 0.84, Teacher’s Emotional Support comprising 4 elements and exhibiting a reliability coefficient of *α* = 0.84, and Classroom’s Mutual Respect constituted by 2 elements with a reliability coefficient of *α* = 0.71. Each of these elements was evaluated using a 5-point Likert scale, enabling participants to express their perceptions, ranging from 1 (strongly disagree) to 5 (strongly agree). To ensure the scale’s reliability in the current context, we conducted a reliability analysis and found the overall Cronbach’s alpha for the scale to be 0.89, further supporting its internal consistency.

#### Growth mindset

Participants’ beliefs regarding the malleability of intelligence were assessed using three items from the Dweck Mindset Instrument (Dweck, 2006). Each item prompted individuals to express their agreement or disagreement with the concept of intelligence being changeable, utilizing a 6-point Likert scale ranging from 1 (strongly disagree) to 6 (strongly agree). The Chinese version of this scale was previously validated with Chinese samples, confirming its sound psychometric properties (Zhao et al., 2021). In our study, we calculated Cronbach’s alpha for this scale, which was 0.79, suggesting acceptable internal consistency.

#### Student engagement

Student engagement was evaluated by 10 items adapted from Skinner et al. (2009). These items encompassed two distinct dimensions: behavioral and emotional engagement, with five items for each dimension. A sample item is “e.g., “*I enjoy learning new skills/knowledge in PE class*.” The validity of these constructs was supported by confirmatory factor analysis, which indicated an acceptable fit. Additionally, we assessed the internal consistency of the scale and obtained a Cronbach’s alpha of 0.87, further confirming its reliability in the current study.

### Data analysis

The data analysis process undertaken in this study was multifaceted, designed to delve into the interrelationships among the variables and rigorously assess the research hypotheses. Initially, the exploration commenced with a thorough examination, encompassing both descriptive and correlation analyses conducted using the statistical software SPSS version 28.0. These initial steps were vital in elucidating the characteristics and interconnections among the variables under investigation.

To assess the research hypotheses, Structural Equation Modeling (SEM) was harnessed, employing the Amos program (version 26.0). The analytical journey embarked with the meticulous evaluation of the measurement model, a crucial step to ascertain the construct validity of the scales employed in the study. Subsequently, the structural model was scrutinized to investigate the postulated relationships between the variables, offering an in-depth exploration of both direct and indirect effects within the theoretical framework.

The evaluation of the hypothesized model’s overall adequacy was enriched by the deployment of various fit indices. The examination included a critical assessment of the *χ*^2^-goodness of fit to degree of freedom (df) ratio, with values falling below 3 deemed indicative of a satisfactory model fit. Additional scrutiny was performed through the examination of the Goodness of Fit Index (GFI) and the Comparative Fit Index (CFI), with values equal to or exceeding 0.90 signifying a well-fitting model.

In the quest for a comprehensive understanding of model fit, careful consideration was given to the Root-Mean-Square Error of Approximation (RMSEA) and the Standardized Root-Mean-Square Residual (SRMR) as pivotal fit indices. Typically, an RMSEA value below 0.08 and an SRMR value less than 0.10 are recognized as hallmarks of a robust model fit, consistent with established criteria outlined in the literature (Hu and Bentler, 1999; Marsh et al., 2004).

## Results

Prior to delving into the proposed model examination, a stringent data screening process was meticulously conducted using SPSS 28, adhering to established procedures (Tabachnick et al., 2013). Multiple facets, including missing data, normality, and outlier detection, were rigorously examined to ensure the robustness of subsequent analyses. Addressing missing data, a critical concern in data analysis, involved employing the Expectation–Maximization (EM) algorithm, recognized for its effectiveness in handling missing data, particularly in scenarios with limited sample sizes or substantial missing data points (Kline, 2011). The EM technique proficiently imputes estimated values for missing data, preserving dataset integrity.

Subsequently, a thorough evaluation of normality was conducted utilizing skewness and kurtosis indices. Items exhibiting values surpassing the ±2.0 threshold, indicative of non-normal distribution, were systematically removed to uphold the analytical rigor of our investigation (Kline, 2011). Furthermore, both individual and multiple outliers were carefully examined and managed by employing *Z*-scores for univariate cases and Mahalanobis *D*^2^ for multivariate instances (Tabachnick et al., 2013). The systematic removal of identified outliers was performed to maintain dataset integrity and analytical rigor.

To ensure the construct validity of our measurement models, Confirmatory Factor Analysis (CFA) was employed following meticulous data screening. Various goodness-of-fit indices were utilized to evaluate model suitability. Initially, measurement models for latent constructs (classroom climate, growth mindset, achievement goal orientation, and student engagement) were assessed. Suboptimal model fits were encountered initially.

In response, specific adjustments were implemented to enhance model alignment with the data. This involved removing three items with factor loadings below 0.40 and incorporating two correlational paths linking error terms of two latent constructs. Following these adjustments, the final measurement models exhibited commendable fits with the data, as detailed in Table 1. A comprehensive presentation of descriptive statistics and correlations for all variables is available in Table 2.

**Table 1:** Measurement model indices.

Latent variable	*χ*^2^	df	*χ*^2^/df	CFI	TLI	RMSEA
Classroom climate	244.12	103	1.87	0.96	0.96	0.05
Growth mindset	62.03	30	2.01	0.93	0.92	0.06
Achievement goal	103.15	54	1.89	0.95	0.94	0.05
Student engagement	245.19	108	2.26	0.92	0.91	0.07

**Table 2:** Descriptive statistics and correlations.

Variable	*M*	SD	1	2	3	4
1. Classroom climate	4.12	0.63	1.00			
2. Growth mindset	3.85	0.71	0.26*	1.00		
3. Achievement goal	4.03	0.65	0.41**	0.52**	1.00	
4. Student engagement	4.18	0.68	0.32**	0.46**	0.45**	1.00

*p < 0.05, **p < 0.01.

As shown in Table 2, the descriptive statistics for the key variables of the study are presented, including means and standard deviations. The mean score for classroom climate was *M* = 4.12 (SD = 0.63), indicating a generally positive perception of the learning environment. Growth mindset demonstrated a mean of *M* = 3.85 (SD = 0.71), reflecting moderate to high endorsement of malleable intelligence beliefs among participants. Achievement goal orientation showed a mean of *M* = 4.03 (SD = 0.65), suggesting a strong orientation toward mastery and achievement-related goals. Finally, student engagement had a mean of *M* = 4.18 (SD = 0.68), indicating high levels of engagement among the participants.

The correlation analysis demonstrated that classroom climate was positively correlated with growth mindset (*r* = 0.26, *p* < 0.05), achievement goal orientation (*r* = 0.41, *p* < 0.01), and student engagement (*r* = 0.32, *p* < 0.01). Growth mindset was significantly correlated with achievement goal orientation (*r* = 0.52, *p* < 0.01) and student engagement (*r* = 0.46, *p* < 0.01). Similarly, achievement goal orientation was positively correlated with student engagement (*r* = 0.45, *p* < 0.01). These findings highlight the interconnections between these key constructs, underscoring their roles as potential correlates of student engagement.

The assessment of convergent validity involved a thorough examination that considered factor loadings, CR, and AVE for each construct (Hair et al., 2019). In Table 3, the displayed factor loadings exceed the recommended threshold of 0.70, signifying robust connections between the items and their respective constructs. Additionally, CR values surpassing 0.70 affirm strong internal consistency and reliability within each construct (Fornell and Larcker, 1981). Moreover, AVE values exceeding 0.50 across all constructs provide evidence supporting convergent validity, indicating that over half of the variance in the items correlates with their underlying constructs (Hair et al., 2019).

**Table 3:** Convergent validity.

Construct	Factor loading (*λ*)	Composite reliability (CR)	Average variance extracted (AVE)
Classroom climate	[0.71–0.87]	0.86	0.72
Growth mindset	[0.75–0.91]	0.91	0.81
Achievement goal	[0.73–0.89]	0.85	0.74
Student engagement	[0.72–0.88]	0.89	0.80

Discriminant validity was assessed by comparing the square root of AVE for each construct with inter-construct correlations, as detailed in Table 4 (Fornell and Larcker, 1981). Remarkably, all correlations between constructs consistently fell below the respective square roots of AVE, affirming robust discriminant validity (Hair et al., 2019). This observation underscores the distinctiveness of the constructs and their minimal interrelatedness. For example, while the square root of AVE for classroom climate is 0.85, its correlations with growth mindset, achievement goal, and student engagement are 0.29, 0.40, and 0.36, respectively. Notably, each correlation is considerably lower than 0.85, highlighting the robust discriminant validity of classroom climate (Fornell and Larcker, 1981). Similar trends persist for growth mindset, achievement goal, and student engagement, affirming their distinctiveness and limited inter-correlation (Hair et al., 2019).

**Table 4:** Discriminant validity.

Construct	Square root of AVE	Correlation with other constructs
Classroom climate	0.85	0.29, 0.40, 0.36
Growth mindset	0.90	0.28, 0.39, 0.34
Achievement goal	0.86	0.30, 0.41, 0.38
Student engagement	0.89	0.31, 0.43, 0.40

AVE, average variance extracted.

After confirming the adequacy of the measurement model, we moved forward to analyze different structural models to investigate our research hypotheses. Initially, we contrasted the presumed partial mediation model with the complete mediation model. The complete mediation model involved setting all connections from the predictor variables to the outcome variable to zero. Furthermore, we investigated another model, a direct alternative, where all connections to and from the mediator variable were restricted to zero.

The results of these model comparisons are summarized in Table 5. It showcases the fit indices for each of the evaluated models, including the Direct Effect Model, Full Mediation Model, and Partial Mediation Model. Notably, the chi-square test statistics (*p* < 0.001) indicate significant differences in model fit. The incremental change in chi-square values (Δ*χ*^2^) serves as a critical indicator of improvement or deviation in model fit across the evaluated models.

**Table 5:** Results of fit indices of alternative models.

Model	*χ*^2^	df	Δ*χ*^2^	GFI	CFI	RMSEA	TLI	SRMR
Direct effect model	1421.98 **	628	–	0.80	0.91	0.07	0.89	0.18
Full mediation model	1317.57 **	624	104.41	0.82	0.94	0.05	0.92	0.07
Partial mediation model	1238.22 **	621	79.35	0.84	0.96	0.04	0.95	0.06

**p-value < 0.001, Δ*χ*^2^ = difference in chi-square values between the model and the subsequent model.

**Figure 2:**
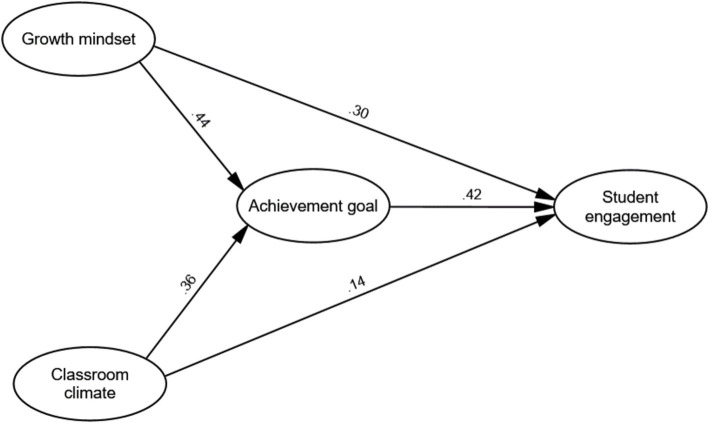
The mediation model.

Interestingly, the Partial Mediation Model demonstrated superior fit indices compared to the other models, as highlighted in the table. The final partially mediated model’s path and parameter estimates are visually represented in Figure 2. It was observed that all path coefficients within the model achieved statistical significance. Notably, the path linking classroom climate to student engagement displayed a relatively modest coefficient. To probe the potential mediating role of achievement goal amidst the study variables, we employed the approach outlined by Baron and Kenny (1986). This analytical method was chosen to discern the intermediary influence of achievement goal in the relationships under investigation. The structural model’s path estimates are summarized in Table 6. This table displays the standardized path coefficients along with their respective t-values for three different models: Direct Effects Model, the Full Mediation Model, and the Partial Mediation Model.

**Table 6:** Path estimates of structural model.

	Standardized path coefficients (*t*-value)		
	Direct effects model	Full mediation model	Partial mediation model
Growth mindset → SE	0.410 (5.22***)		0.302 (3.78**)
Classroom climate → SE	0.218 (2.78**)		0.145 (1.89*)
Growth mindset → achievement goal		0.405 (5.47***)	0.442 (5.61***)
Classroom climate → achievement goal		0.343 (4.46***)	0.368 (4.72***)
Achievement goal → SE		0.499 (6.88***)	0.426 (5.37***)

SE, student engagement. Significance levels are denoted by asterisks: ***p < 0.001, **p < 0.01, *p < 0.05.

These models elucidate the relationships among growth mindset, classroom climate, achievement goal orientation, and student engagement (SE) within the structural model. Baron and Kenny’s (1986) method was employed to explore the mediating role of achievement goal orientation in the relationship between classroom climate and student engagement. Prior to conducting the mediation analysis, the conditions stipulated by Baron and Kenny were methodically ensured.

As indicated in Table 6, the Direct Effects Model demonstrates significant path coefficients, revealing positive relationships between growth mindset and student engagement (*β* = 0.410, *t* = 5.22, *p* < 0.001), as well as between classroom climate and student engagement (*β* = 0.218, *t* = 2.78, *p* < 0.01). Similarly, significant paths are observed between growth mindset and achievement goal orientation (*β* = 0.405, *t* = 5.47, *p* < 0.001), as well as between classroom climate and achievement goal orientation (*β* = 0.343, *t* = 4.46, *p* < 0.001). Moreover, a substantial relationship is evident between achievement goal orientation and student engagement (*β* = 0.499, *t* = 6.88, *p* < 0.001).

In the Full Mediation Model, the paths from growth mindset and classroom climate to student engagement diminish in magnitude when achievement goal orientation is included as a mediator. Specifically, while the path from growth mindset to student engagement remains significant (*β* = 0.302, *t* = 3.78, *p* < 0.01), the relationship between classroom climate and student engagement becomes non-significant (*β* = 0.145, *t* = 1.89, *p* > 0.05).

Contrarily, in the Partial Mediation Model, where achievement goal orientation acts as a mediator between classroom climate and student engagement, both the direct paths from classroom climate to student engagement (*β* = 0.145, *t* = 1.89, *p* < 0.05) and the indirect path mediated by achievement goal orientation maintain significance (*β* = 0.426, *t* = 5.37, *p* < 0.001). This suggests a partial mediation effect, demonstrating the interplay between classroom climate, achievement goal orientation, and ultimately, student engagement. Thus, the results portrayed in Table 6 substantiate the mediation role of achievement goal orientation in the relationship between the constructs in the structural model.

## Discussion

This study aimed to investigate the complex interconnections among classroom climate, achievement goal orientations, growth mindset, and student engagement in EFL education. The research outcomes shed light on the substantial correlations between these elements, adding valuable insights to the educational psychology field. By delving into the intricate relationships among these factors, this study contributes to the existing literature, enriching our understanding of their interplay in the educational context.

As hypothesized (H1), a direct and positive relationship was found between growth mindset and student engagement. This outcome aligns with the conceptual framework of Dweck’s mindset theory (2006), emphasizing that a growth mindset, which centers on the belief in the potential for intelligence and abilities to grow through effort, positively correlates with enhanced student engagement (Derakhshan et al., 2022; Karlen et al., 2019; Li, 2023; Tseng et al., 2020; Zeng et al., 2016; Zhao et al., 2021). In the EFL context, where learners frequently encounter linguistic and cultural barriers, a growth mindset encourages students to view these challenges as opportunities to improve rather than obstacles (Derakhshan et al., 2022; Li, 2023).

The reason for this connection is rooted in the resilience and persistence that a growth mindset fosters. Students with this mindset are more likely to tackle difficulties, apply effective learning strategies, and maintain a positive outlook toward their language studies (Claro et al., 2016). By reducing fear of failure, a growth mindset helps students take risks and engage more fully in class activities (Tseng et al., 2020; Zeng et al., 2016). Moreover, these students often set mastery-oriented goals, emphasizing personal growth and skill development, which strengthens their engagement and academic performance (Rhew et al., 2018; Cheng, 2023). In addition, a growth mindset is closely linked to intrinsic motivation—learning driven by internal rewards such as personal satisfaction. Students who are intrinsically motivated tend to be more deeply involved with their learning materials, seek out additional resources, and continue working through challenges. This persistence enhances sustained engagement in EFL learning (Derakhshan et al., 2022; Zhao et al., 2021).

Furthermore, the results support the hypothesized direct relationship between classroom climate and student engagement (H2). A supportive and respectful classroom environment, characterized by mutual respect, teacher support, and collaboration, has been shown to significantly influence students’ emotional well-being and commitment to learning (Fraser, 1989; Joe et al., 2017; Wang et al., 2020). This finding aligns with previous research emphasizing the importance of fostering a positive classroom atmosphere to cultivate student engagement in EFL classrooms (Li, 2024; Mohammad Hosseini et al., 2022; Namaziandost et al., 2024). The key reason for this relationship is that a supportive classroom environment fosters a sense of safety and belonging. When students feel respected and valued, they are more inclined to participate actively in class activities, take intellectual risks, and invest more effort in their language learning (Aldridge et al., 2020; Conchas, 2001; Liu and Geng, 2023). Emotional support from the classroom environment helps reduce anxiety, which is particularly important in EFL settings, where students often face language barriers and cultural adjustments (Liu and Geng, 2023).

Additionally, a positive classroom climate encourages collaboration and peer support, which are vital for language learning. In an environment where students work together and support one another, they can practice language skills more openly and learn from their peers’ strengths (Fraser, 1989; Joe et al., 2017). This cooperative atmosphere not only improves linguistic competence but also strengthens students’ motivation to engage with both the learning materials and their classmates (Wang et al., 2020). Teachers are instrumental in shaping the classroom climate. By fostering an inclusive, respectful, and collaborative environment, educators can enhance students’ engagement with both the subject matter and each other. Clear communication, constructive feedback, and opportunities for teamwork all contribute to a positive classroom atmosphere that supports student involvement (Li, 2023; Wang et al., 2020; Namaziandost et al., 2024).

Our findings also provided strong support for the mediating role of achievement goal orientations in the relationship between growth mindset and student engagement (H3). This result is consistent with Achievement Goal Theory (Ames, 1992), indicating that a growth mindset, which encourages mastery-oriented goals focused on learning and skill development, influences student engagement positively (Gonida et al., 2009; Lazarides and Rubach, 2017; Mädamürk et al., 2021; Miller et al., 2021). Specifically, students with a growth mindset are more likely to set goals that emphasize personal growth, effort, and self-improvement rather than seeking external validation (Rhew et al., 2018; Cheng, 2023). These mastery goals, in turn, promote deeper learning strategies, persistence, and resilience, all of which are closely linked to higher levels of student engagement (Alhadabi and Karpinski, 2020).

This relationship is further supported by Wolters and Hussain (2015), who found that students with mastery-oriented goals exhibit greater behavioral engagement and are more likely to view challenges as opportunities for growth. Additionally, our study builds on the work of Bai and Wang (2023), demonstrating that a combination of growth mindset, self-efficacy, and intrinsic value significantly predicts academic success in EFL learners. The mediating role of achievement goal orientations underscores the importance of fostering mastery goals in language learning contexts to enhance engagement and support long-term academic success. The reason for this mediation is that mastery-oriented goals drive students to engage more deeply with their learning, even when facing difficulties. In EFL contexts, where language learning can be particularly challenging, students focused on mastery goals are more likely to apply effective strategies, seek help when needed, and stay motivated through setbacks. This focus on personal development not only boosts engagement but also improves academic performance, as students remain committed to their learning despite obstacles.

Similarly, the study revealed that achievement goal orientations mediated the relationship between classroom climate and student engagement, supporting H4. Previous studies have shown that a supportive and autonomy-enhancing classroom environment fosters mastery-oriented goals in students (Ames, 1992; Jiang and Zhang, 2021; Madjar et al., 2019; Patrick et al., 2011; Rinehart and Espelage, 2016; Shi et al., 2023). In turn, mastery goals promote intrinsic motivation, resilience, and deeper learning strategies, which are key drivers of student engagement (Pintrich, 2000; Huang, 2016; Lazarides and Rubach, 2017; Mädamürk et al., 2021; Miller et al., 2021). Our results align with Wang et al. (2023), who found that classroom environments that emphasize mastery over performance promote greater engagement and academic success in EFL learners. The mediating role of achievement goal orientations highlights that classroom climate shapes engagement not only through emotional and social support but also by influencing the cognitive and motivational approaches students adopt. When students see their classroom as supportive and collaborative, they are more likely to adopt mastery goals, focusing on personal improvement and learning.

The deeper reason for this mediation is that a positive classroom climate encourages students to set meaningful learning goals. In such environments, students are more willing to engage actively in language tasks, take intellectual risks, and persist through challenges. A supportive atmosphere reduces anxiety and self-doubt, allowing students to concentrate on mastering the language without the fear of failure. This combination of a positive classroom climate and mastery-oriented goals leads to higher engagement and improved academic outcomes.

In conclusion, this study’s findings underscore the significance of growth mindset, classroom climate, and achievement goal orientations in predicting student engagement within EFL educational contexts. The interplay among these factors elucidates the complex dynamics that contribute to student engagement and highlights potential avenues for educational interventions aimed at enhancing student engagement in EFL learning environments. Future research could explore specific strategies and interventions that leverage growth mindset and foster a positive classroom climate to further enhance student engagement and learning outcomes in EFL contexts.

## Conclusion

Overall, this research has unveiled the intricate relationships that exist among growth mindset, classroom climate, achievement goal orientations, and student engagement within the context of EFL learning. The findings demonstrated that a growth mindset and a positive classroom climate are both significantly related to student engagement, which aligns with prior research highlighting their pivotal roles in motivating learners and shaping the educational environment. Furthermore, this study revealed that achievement goal orientations mediate these relationships. A growth mindset fosters mastery goal orientations, and a positive classroom climate encourages the adoption of these orientations. In turn, mastery goal orientations have a direct and positive impact on student engagement. This mediation effect highlights the importance of considering students’ achievement goals and the learning environment when aiming to enhance their engagement in EFL learning.

The findings from this study carry substantial implications for educators, administrators, and policymakers engaged in EFL education. Firstly, educators should prioritize cultivating a growth mindset among EFL learners. Implementing strategies within the curriculum that promote beliefs in the malleability of language proficiency is essential. Encouraging learners to perceive language acquisition as a journey marked by challenges, where effort and resilience are pivotal to success, can significantly foster a growth mindset. Secondly, the creation of a positive and supportive classroom climate must be a primary focus. Educators and school administrators should allocate resources toward establishing an environment characterized by respect, safety, and inclusivity. Initiatives promoting positive teacher-student and peer relationships, clear behavioral expectations, and a sense of belonging can effectively contribute to a nurturing classroom climate.

Moreover, educators hold a crucial role in shaping students’ achievement goal orientations. By fostering a supportive learning environment that emphasizes mastery goals, educators can indirectly influence student engagement. Prioritizing interventions aimed at enhancing classroom climate should consider the mediating role of achievement goal orientations. Encouraging a positive classroom climate can pave the way for the adoption of mastery goals, thereby potentially increasing student engagement levels. These implications underscore the pivotal role of educators and administrators in shaping both the mindset and learning environment conducive to maximizing student engagement in EFL education.

While this investigation offers valuable insights into the connections among growth mindset, classroom climate, achievement goal orientations, and student engagement within the realm of EFL learning, it’s crucial to acknowledge several constraints. To start, the research design operates on a cross-sectional basis, making it arduous to establish definitive cause-and-effect relationships among the variables. Future studies should contemplate adopting longitudinal methodologies to scrutinize the evolving dynamics among these factors over time.

Secondly, this study concentrates on a specific educational context, specifically EFL learning, and might not be directly applicable to other language learning environments or diverse educational settings. Further research is imperative to scrutinize whether analogous patterns and mediating influences exist across various language learning contexts and among students from diverse age groups. Lastly, this study relied on self-reported measures, which are susceptible to potential biases, such as social desirability. Subsequent research could benefit from integrating diverse data sources, such as observations or behavioral indicators, to augment the validity and credibility of the outcomes.

## Data Availability

The raw data supporting the conclusions of this article will be made available by the authors, without undue reservation.
